# Prevalence and Molecular Epidemiological Data on *Dirofilaria immitis* in Dogs from Northeastern States of India

**DOI:** 10.1155/2015/265385

**Published:** 2015-01-21

**Authors:** Sonjoy Kumar Borthakur, Dilip Kumar Deka, Saidul Islam, Dilip Kumar Sarma, Prabhat Chandra Sarmah

**Affiliations:** ^1^College of Veterinary Sciences & A.H., Central Agricultural University, Selesih, Aizawl, Mizoram 796 014, India; ^2^College of Veterinary Science, Assam Agricultural University, Khanapara, Guwahati, Assam 781 022, India; ^3^National Research Centre on Pigs (ICAR), Rani, Guwahati, Assam 781 131, India

## Abstract

The aim of the present study was to determine the prevalence of *Dirofilaria immitis* in stray, pet, and working dogs (*n* = 413, 266, and 103, resp.) from Guwahati (Assam) and Aizawl (Mizoram), areas located in two Northeastern States of India. Diagnostic methods applied were microscopy (wet film and Knott's concentration technique), immunological test (Ag ELISA by SNAP 4Dx ELISA kit), and molecular tools (polymerase chain reaction and sequencing), which evidenced 11.38, 18.03, and 13.93% of positive animals, respectively. No significant differences were observed by area (18.23% versus 17.68%) nor by sex (18.1% versus 17.9%), whereas stray dogs proved more infected than other groups (*P* < 0.05). ELISA test evidenced an overall 22.69% of occult infections, mainly in working dogs (60%), and molecular techniques detected *Dirofilaria (Nochtiella) repens* in 4 stray dogs from Guwahati. Characterization of *D. immitis* isolates for ITS-2 region showed close identity with South Asian isolates.

## 1. Introduction

*Dirofilaria immitis*, the heartworm of dog, is one of the most important filaroid nematodes responsible for causing canine dirofilariosis. Heartworm inhabits the right ventricle and pulmonary arteries of dogs and other animals. The heartworm parasite is transmitted by various mosquitoes belonging to the genera* Culex*,* Aedes*, and* Anopheles*. Adult female* D. immitis* lays microfilariae, which are taken up by suitable mosquito vectors and subsequently develop to the infective 3rd larval stage. Transmission takes place when a potential vector bites dogs or other hosts during a subsequent blood meal. It takes about 6-7 months to become an adult stage. Pathophysiological response to heartworm infection is mainly due to the presence of adult worm. The main clinical symptoms in dirofilariosis include persistent cough, difficult breathing, and poor exercise tolerance followed by ascites, anorexia, and weight loss. The symbiotic relationship with* Wolbachia *(a rickettsia) along with* D. immitis* amplifies disease severity [1]. The pathogenesis, pathology, and clinical manifestations of heartworm have been aptly reviewed [2].

Laboratory diagnosis of dirofilariosis in live animals is always in forefront in terms of demonstration and identification of microfilariae in tested blood sample. Radiography and cardiography aid in the diagnosis of* D. immitis*, but confirmatory and reliable diagnosis for heartworm disease is dependent on serology and molecular tests. Sometimes, in circulating blood of heartworm infected dogs microfilariae are absent and such condition is termed as “occult infection.” In this case, obviously microscopy and PCR give false negative results. Several commercial ELISA based test kits are available to diagnose heartworm in dogs but these kits are not widely used in India. DNA based techniques provide an alternative approach which is very sensitive and accurate for identification of the filarial parasites [3].

Heartworm disease due to* D. immitis* has also been reported as an emerging zoonosis by several authors [4–8]. Human infection is mostly located in temperate, tropical, and subtropical areas of the world. So far, more than 1,700 human cases of dirofilariosis (including >370 pulmonary cases) have been documented worldwide, suggesting that wherever canine dirofilariosis is present humans are at risk of infection [9, 10]. Most* D. immitis* human infections are asymptomatic showing typical coin lesions on chest radiography and are often mistakenly removed as neoplasm [11]. In India, the first case of human pulmonary dirofilariosis due to* D. immitis* was reported from Mumbai [12]. After that, several cases of human dirofilariosis have been reported in India [13].

In animals, there have been both epidemiological and clinical case studies of this worm worldwide [14–20]. Prevalence of this parasite in dogs from several parts from India has been reported [21, 22]. Limited works revealed occurrence of 33.75%* D. immitis* in Mizoram, a Northeastern State of India, on the basis of examination of 240 dogs at slaughter [23]. A recent study, based on wet film and antigen tests, revealed 4.76 to 29.54% prevalence of* D. immitis* in a hospital population of dogs from Assam [24]. But, no systematic work on epidemiological aspect of* D. immitis* has been carried out in the northeastern part of this country using combination of conventional, serological, and molecular diagnostic techniques.

The importance of this parasite and the paucity of information about the prevalence of canine heartworm in the Northeastern States of India inspired us to conduct the present plan of work. We studied blood samples from dogs that presented for veterinary attention and from dogs captured by various nongovernmental organizations and maintained in kennels. The prevalence was studied by means of conventional microscopy, an immunological test, and molecular techniques, and their efficacy was compared. Further, we explored some of the molecular characteristics of* D. immitis* in the studied area.

## 2. Materials and Methods

### 2.1. Study Areas

**Figure 1:**
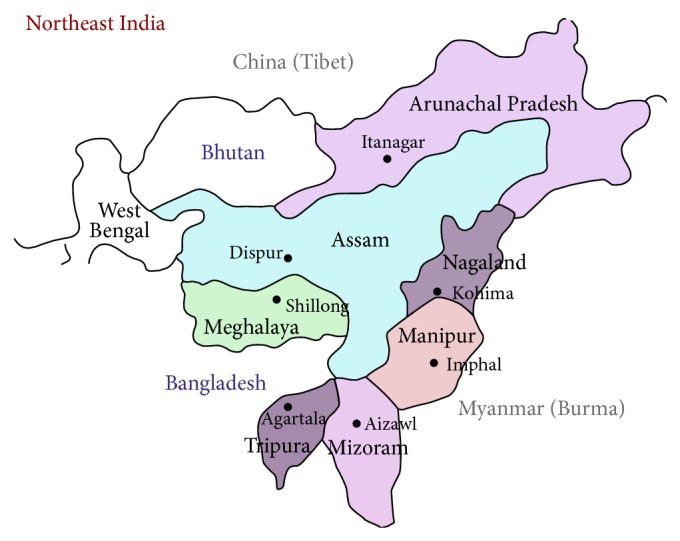
Map showing the study areas.

The study was undertaken systematically for a period of one calendar year from August 2011 to July 2012, in dogs from Guwahati and Aizawl. Guwahati, a city of Assam having annual rainfall of 1500–2600 mm, is located at 26°11′0′′ latitude N and 91°44′0′′ longitude E, and Aizawl, the capital city of Mizoram State having annual rainfall of 2400–2962 mm, is located at 23°43′27′′N and 92°43′2′′E. The cities are separated by surface distance of 550 km. The location of epidemiological study undertaken for the present study is shown in the map (Figure 1).

### 2.2. Selection of Dogs

In the present investigation, 3 categories of dogs grouped as working dogs of military and paramilitary force, pet dogs, and stray dogs were selected. Pet dogs of different breeds and paramilitary dogs mostly of Labrador and German Shepherd breeds brought to the Teaching Veterinary Clinical Complexes (TVCC) of the College of Veterinary Science, Assam Agricultural University, Khanapara, Guwahati, Assam, and the College of Veterinary Sciences & A.H., Central Agricultural University, Selesih, Aizawl, Mizoram, during the study period were taken for the study. In case of military dogs, most of the blood samples were directly sent to the Department of Parasitology for routine check-up of heartworm infection. The stray dog population consisted of local nondescript street dogs of either sex captured from different parts of the city for sterilization by local nongovernmental organizations like Peoples for Animal (PFA) and Just Be Friendly (JBF).

Three categories of dogs like working (103), pet (266), and stray (413) totaling 782 numbers were examined. Dogs under the study were of both sexes and a total of 488 dogs from different localities of Guwahati and 294 dogs from Aizawl formed the entire base of study during the programme.

### 2.3. Blood Sampling

Approximately 5 mL of blood was drawn from the cephalic vein collected in disodium salt of ethylene diamine tetraacetic acid (Na_2_EDTA) vacuum tubes and stored at 4°C until further use.

### 2.4. Parasitological Investigation

The prevalence study for* D. immitis* was conducted on the basis ofconventional, immunological, and molecular techniques. The conventional tests were based on microfilarial availability in blood samples assessed on the basis of wet blood film method and modified Knott's concentration technique (KCT) [25]. Subsequently microfilarial identification was done [26]. The immunological evidence was based on the presence of heartworm antigens in tested blood samples and was performed with a commercially available ELISA test kit (SNAP 4Dx) following manufacture's test protocol. Molecular evidence was based on amplification of worm targeted DNA. Blood samples negative for* D. immitis* circulating antigen but positive at KCT were processed for this technique. Fresh adult* D. immitis* and positive blood samples collected from Aizawl slaughter house were used to standardize techniques and used as positive control for the tests. In addition, blood samples were taken from dogs clinically infected and found positive for* D. immitis* being confirmed by KCT and SNAP tests. Briefly, the molecular technique for the present study was performed as follows.

### 2.5. Isolation of Genomic DNA from Blood and Adult Parasites

Isolation of genomic DNA from blood and adult parasite was carried out using the DNeasy Blood and Tissue Kit (Qiagen Kit, Catalogue number 69504) as per the protocols provided by the manufacturer. The final templates were kept at −20°C.

### 2.6. PCR Assays

PCR technique targeted to amplify the ITS-2 region of filarial worms' rDNA was applied according to previously described protocols [3, 27]. Briefly, reaction mixture comprised 2.5 *μ*L Taq polymerase buffer (10x), 01 *μ*L dNTP (10 mM), 0.5 *μ*L MgCl_2_ (50 mM), 0.75 *μ*L of each forward and reverse primer (60 pM), 1 U Taq polymerase, and 3.0 *μ*L template DNA (60 ng/*μ*L concentration), by making the final volume up to 25.0 *μ*L with NFW. The cycling condition used for amplifying the targeted product consisted of an initial denaturing step at 94°C for 2 min and 32 cycles of denaturing (30 s at 94°C), annealing (30 s at 60°C for 5.8S-ITS-2-28S-based primers and 30 s at 58°C for ITS-2-based primers), and extension (30 s at 72°C); a final extension (7 min at 72°C or 12 min at 72°C for cloning); and a soak at 4°C in a Techne-5000 thermal cycler (Bibby Scientific). The confirmation of the amplified products was made by gel electrophoresis of the PCR product in 1.5% agarose gel stained with ethidium bromide and visualized under gel doc (DNR Bio-Imaging System, MiniLumi).

The specificity of the PCR amplification for the corresponding* D. immitis* target, both on representative positive blood samples and on adult worms, was assessed by amplicon purification followed by cloning and sequencing.

### 2.7. Cloning of the Genomic Region

Cloning of the PCR amplicon(s) for the genomic region as described above has been performed using pDrive cloning vector (Qiagen PCR Cloning Kit, Catalog number 231124). DH5*α E. coli* cell was used for transformation of the plasmid using Transform Aid Bacterial Transformation Kit (Fermentas, Catalog number K2711). Subsequently, clones were confirmed by clones' confirmation PCR.

### 2.8. Sequencing and Analysis of ITS-2

The recombinant clones were sent to the Department of Biochemistry, University of Delhi, South Campus, for automated sequencing. The sequences obtained were aligned and compared with other published sequences of* D. immitis* of dogs by Clustal W method using DNASTAR software and phylogenic analysis was done. Sequences were compared in silico with sequences of* D. immitis *(ITS-2) rDNA available in the gene bank for each gene examined using the nucleotide-nucleotide “Basic Local Alignment Search Tool.” Sequences were submitted to NCBI to obtain accession numbers.

## 3. Results

**Figure 2:**
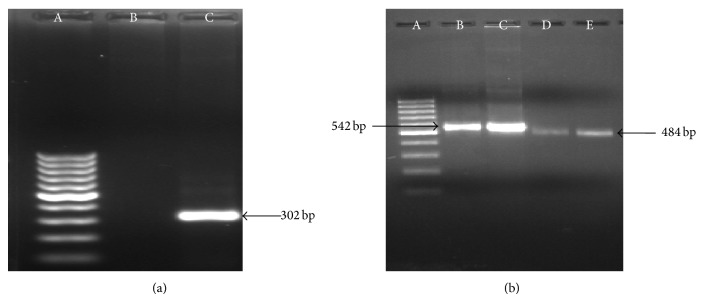
(a) Gel picture showing amplification of* D. immitis* (specific primers). Lane A: 100 bp ladder. Lane B: negative. Lane C: PCR product of ITS-2. (b) Gel picture showing amplification of* D. immitis* and* D. repens* (panfilarial primers). Lane A: 100 bp ladder. Lanes B and C: amplification for* D. immitis*. Lanes D and E: amplification for* D. repens*.

The study of the prevalence of canine heartworm, carried out by means of different techniques, obviously produced different results. Overall 6.26% (49/782) blood samples analysed proved microfilaraemic under wet film methods, whereas infection rates found by means of the other techniques were higher. As expected, the highest number of positive animals was detected by the ELISA test, which evidenced an overall prevalence of 18.03% (Table 1), without differences by area and by sex. On the contrary, in both areas differences were evidenced by group of dogs.  Table 2 summarizes data on the efficacy of KCT, ELISA test, and PCR, which classified as infected 11.38%, 18.3%, and 13.93% of the examined dogs, respectively, with stray dogs always more infected than other groups. Moreover, PCR analysis with primers specific for* D. immitis *detected in 88 animals the 302 bp expected band (Figure 2(a)) whereas that with panfilarial primers confirmed the presence of* D. immitis* DNA (band of 542 bp) in 88 dogs and recognized* D. repens* DNA (band of 484 bp) in further 4 animals (Figure 2(b)). The present study also revealed overall 22.69 percent occult cases which were determined on the basis of differences between heartworm positive cases in PCR test and antigen detection test (SNAP 4Dx). The working dogs had the highest occult infection (60%) followed by pet (29.16%) and stray (17.75%) dogs.

**Figure 3:**
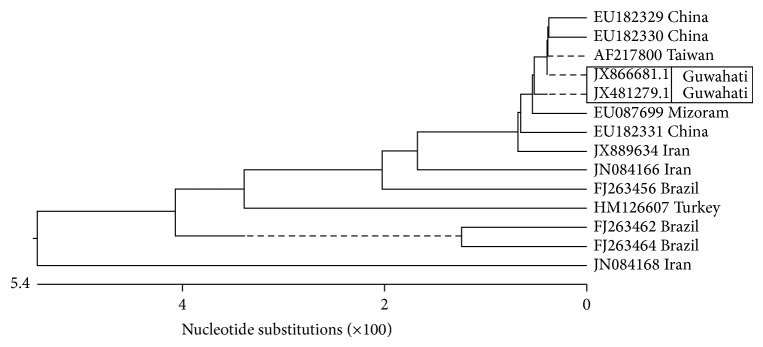
Phylogenetic tree constructed for* Dirofilaria immitis* from the ITS-2 region using Clustal W of DNASTAR.

Phylogenetic analysis of two Guwahati isolates of* Dirofilaria immitis* was comparedwith additional twelve sequences from the NCBI GenBank by Clustal W of DNASTAR. Sequences from isolates of India (EU087699), Taiwan (AF217800), China (EU182329, EU182330, and EU182331), Iran (JX889634, JN084166, and JN084168), Brazil (FJ263456, FJ263464, and FJ263462), and Turkey (HM126607) were included. All the sequences fell under the same group; however, the sequences from Southeast Asia were more closely related. The phylogenetic tree constructed based on this finding is depicted (Figure 3). Pairwise distance analysis of the ITS-2 sequences of Guwahati isolates showed 84.7 to 99.8% identity and the divergence ranged from 0.0% (Taiwan, AF217800) to 13.6% (Iran, JN084168). On the contrary, sequences of Guwahati isolates (JX481279 and JX866681) were 98.6–98.9% identical to that of Taiwan species (AF217800) (Table 3). Accession numbers for each sequence for 2 isolates of* D. immitis* (accession numbers JX481279 and JX866681) and for* D. repens* (accession number JX524743) were obtained from GenBank.

## 4. Discussion

The present study provides the first comprehensive assessment of* D. immitis* infection in dogs from the Northeastern States of India. The number of dogs proven positive for* D. immitis* in one or more diagnostic tests was variable according to the test applied. Recording of 6.26% blood samples microfilaraemic under wet film does not give the actual epidemiological situation of the studied areas owing to the low sensitivity of the method and to the failure in microfilarial species differentiation [28]. Currently, abundant literature suggests detecting antigen test as the most sensitive diagnostic method for canine heartworm [29]. The present study showed prevalence of 9.02%* D. immitis* infection in pet dogs and 25.90% in stray dogs. A similar type of varying from 4.76% to 29.54%* D. immitis* infection was recorded in Assam in pet and street dogs, respectively [24]. The records of highest prevalence in stray/street dogs are likely due to their free roaming habits making them vulnerable to being bitten by different mosquito vectors. Moreover, the present study was carried out in a geographical location where subtropical climate and deciduous forest land prevail, therefore in an environment where high rainfall and humidity create ideal mosquito breeding places.

Our present study also revealed a higher prevalence of* D. immitis* in male dogs, but we could not draw a conclusion on the differences of prevalence amongst male and female dogs. Like most record on heartworm prevalence [24, 30–32] and unlike few cases from elsewhere [33, 34], our study found a nonsignificant higher prevalence of* D. immitis* in male dogs.

In the present investigation, many dogs which were found positive using SNAP 4Dx kit revealed occult infections. The same samples when subjected to PCR studies revealed lesser percent prevalence. Occult infections (amicrofilaraemic infections) could arise due to several causes like low parasite burdens, prepatent infection by young adults, infection of dog by only male worm, geriatric female worm, and immune response from the host against microfilariae or under microfilaricidal therapy. The high percentage of occult infection is not uncommon and was previously reported by several authors from different parts of the world [35, 36]. Higher occult cases recorded in working (60%) and pet (29.16%) dogs in comparison to stray dogs (17.25%) might be due to the fact that owners of pet and working dogs are very much concerned about the health status of their animals. Hence, there is regularity in their health check-up that surely often required administration of anthelmintic drugs like ivermectin, an endectocide drug whose microfilaricidal activity reduces the number of circulating microfilariae. On the other hand, stray dogs are seldom taken care of with such type of medications.

The overall prevalence of* D. immitis* detected by PCR was 13.93% (109/782), lower than that evidenced by ELISA test, probably due to occult infections or to possible failure in DNA extraction. Hence, the main benefit of PCR in epidemiological surveys on dirofilariosis is 100% confirmatory species identification when mixed infections coexist [3, 37]. Detection of 4 dogs infected by* D. repens* supports the reports of human infection in Northeastern States of India [38] and is an alarming finding, since this species, apparently more adapted than* D. immitis* to the human host, often succeeds in its development to adult worm.

Phylogenetic analysis of* D. immitis* isolates of Guwahati showed a close identity with certain South Asian isolates of* D. immitis. *Pairwise homology analysis revealed 98.6–98.9% identity with a few sequences available at NCBI GenBank. Previously, 92.6% homology of* D. immitis* of Mizoram isolate with* D. immitis* of Taiwan isolate (AF217800) was documented [39]. In the present study, between Guwahati and Mizoram isolates, the identity was 95.4 to 95.7% and the divergence was 0.5 to 0.7%.

## 5. Conclusion

This study confirms the predominance of* D. immitis* in the northeastern region of India and reestablishes the area as heartworm endemic. KCT along with antigen ELISA detection test confirmed their sensitivity, whereas molecular techniques confirmed their value in identification of canine filarial worms. The presence of both* Dirofilaria* species should alert physicians to the risk of human infections.

## Figures and Tables

**Table 1:** Prevalence of *Dirofilaria immitis* by category of dogs and area of study on the basis of Ag ELISA test.

Dog category	Guwahati			Aizawl			Overall		
	Numbers	Positive %	Chi value	Numbers	Positive %	Chi value	Numbers	Positive %	Chi value
Stray dogs	223	63 (28.25)	27.6139	190	44 (23.15)	11.0458	413	107 (25.90)	36.7706
Pet dogs	174	17 (9.77)		92	7 (7.60)		266	24 (9.02)	
Working dogs	91	9 (9.89)		12	1 (8.33)		103	10 (9.70)	

Total	488	89 (18.23)		294	52 (17.68)		782	141 (**18.03**)	

**Table 2:** Comparative efficacy percentage of microscopy, Ag ELISA, and PCR in detecting *Dirofilaria immitis* infection in dogs.

		Detection methods			
Dog category	Number of blood samples tested	Microscopy (Knott's technique)	Ag ELISA (SNAP 4Dx test)	PCR	
		Numbers of positive (%)	Numbers of positive (%)	Specific primer for *D. immitis* (%)	Panfilarial primers (%)
Stray	413	69 (16.70%)	107 (25.90%)	88 (21.30%)	92 (88 + 4^*^) (22.27%)
Pet	266	16 (6.01%)	24 (9.02%)	17 (6.39%)	17 (6.39%)
Working	103	4 (3.88%)	10 (9.70%)	4 (3.88%)	4 (3.88%)

Grand total	782	89 (11.38%)	141 (18.03%)	109 (13.93%)	113 (14.45%)

^*^ *Dirofilaria repens*.

**Table 3:** ITS-2 sequence pair distance analysis of *Dirofilaria immitis* isolates compared with homologous isolates (slow/accurate, IUB).

		Percent identity															
		**1**	**2**	**3**	**4**	**5**	**6**	**7**	**8**	**9**	**10**	**11**	**12**	**13**	**14**		
Divergence	**1**		99.8	94.1	91.0	90.2	90.2	84.7	93.4	95.5	95.7	95.9	95.4	94.5	98.9	**1 **	*JX866681.1 * **Guwahati**
	**2**	0.2		94.1	91.0	90.2	90.2	84.7	93.4	95.5	95.4	95.7	95.2	94.3	98.6	**2 **	*JX481279.1 * **Guwahati**
	**3**	5.1	5.1		95.7	94.9	94.1	91.4	93.3	93.7	95.7	88.6	93.7	86.3	93.7	**3**	FJ2634568 Brazil
	**4**	8.7	8.7	4.6		97.6	93.7	89.0	90.2	90.6	92.2	85.5	90.6	83.1	90.6	**4 **	FJ263462 Brazil
	**5**	9.6	9.6	5.5	2.5		95.3	88.3	90.2	89.8	91.4	84.8	89.8	82.4	89.8	**5 **	FJ263464 Brazil
	**6**	9.6	9.6	6.3	6.8	5.0		87.9	89.8	89.5	91.4	84.8	89.5	83.2	89.8	**6 **	HM126607 Turkey
	**7**	13.6	13.6	6.1	8.9	9.3	9.8		88.5	88.5	86.8	80.0	88.1	78.6	84.4	**7 **	JN084168 Iran
	**8**	4.1	4.1	4.3	7.9	7.4	7.9	12.8		97.4	95.4	89.3	97.1	87.6	93.4	**8 **	JN084166 Iran
	**9**	1.8	1.8	3.8	7.4	7.9	8.4	12.7	2.8		98.0	91.6	99.2	89.9	95.5	**9 **	JX889634 Iran
	**10**	0.5	0.7	2.9	6.9	7.3	7.3	13.6	4.1	1.2		92.0	97.0	90.7	95.0	**10 **	EU087699 Mizoram
	**11**	0.0	0.2	5.4	9.3	10.2	10.2	14.4	4.3	1.9	0.5		93.2	95.4	94.8	**11 **	EU182329 China
	**12**	1.2	1.5	3.8	7.4	7.9	8.4	13.2	3.1	0.9	0.7	1.3		91.8	94.8	**12 **	EU182331 China
	**13**	0.7	1.0	6.7	10.5	11.4	10.4	15.5	5.2	2.8	1.3	0.7	2.0		93.4	**13 **	EU182330 China
	**14**	0.0	0.2	5.1	8.7	9.6	9.6	13.7	4.1	1.8	0.5	0.0	1.2	0.7		**14 **	AF217800 Taiwan
		**1**	**2**	**3**	**4**	**5**	**6**	**7**	**8**	**9**	**10**	**11**	**12**	**13**	**14**		
